# Perforated Gallbladder into the Abdominal Wall

**DOI:** 10.1155/2022/4782539

**Published:** 2022-10-13

**Authors:** M. Puglisi, M. Peter, B. Egger

**Affiliations:** Department of Surgery, HFR Fribourg-Cantonal Hospital, Fribourg, Switzerland

## Abstract

**Objective:**

Perforation of the gallbladder (PG) is a dreaded complication of an acute cholecystitis and is associated with increased morbidity and mortality. Cholecystocutaneous abscess (CCA) is an extremely rare complication. There is usually a history of cholecystolithiasis or neglected chronic gallbladder disease. We report a case of perforated gallbladder into the abdominal wall.

**Methods:**

A 65-year-old female, obese, was admitted to our department complaining of right upper quadrant abdominal pain. The diagnosis of acute cholecystitis was based on the clinical picture, laboratory test, and ultrasound findings. She was treated with oral antibiotics for 10 days and readmitted due to a painful, erythematous mass on the right subcostal region. An abdominal computed tomography showed the presence of a subparietal formation in communication with the gallbladder, and a gallbladder perforation was postulated. The treatment consisted of percutaneous drainage of the abdominal wall abscess followed by laparoscopic cholecystectomy in a two-stage protocol. Anatomical pathology analysis found chronic inflammation and excluded malignancy. The postoperative follow-up was uneventful. *Discussion*. This case demonstrates a very rare presentation of PG that created an abscess into the muscles of the abdominal wall. This kind of PG is rarely seen due to medicine improvements. When the conditions of the patient are good, rather than perform immediate surgery that could lead to serious complications, we propose a two-stage approach.

**Conclusion:**

CCA is a possible complication of gallbladder's pathology that all surgeons have to know. There is no standard baseline management for this pathology, due to the few numbers of cases and to the differences in the quality of the patients' illness. We suggest a two-stage approach with drainage of the abscess followed by laparoscopic cholecystectomy with abscess debridement.

## 1. Introduction

Perforation of the gallbladder is a dreaded complication of cholecystitis, and it is associated with increased morbidity and mortality [1]. The perforation could be due to trauma, iatrogenic causes, to carcinoma of the gallbladder or could be spontaneous. Cholecystocutaneous abscess (CCA) is an extremely rare complication secondary to cholecystitis. There is usually a history of cholecystolithiasis or neglected chronic gallbladder disease. In the physiopathology of the development of this condition, there could be repeated episodes of cholecystitis or there could be obstruction of the gallbladder with stone increasing intragallbladder pressure and then compromising gallbladder wall blood's circulation, later resulting in necrosis of the gallbladder wall [2]. In the end, the gallbladder perforates. Once pierced, the gallbladder may drain into the peritoneal cavity or due to adherences into adjacent organs or the abdominal wall [3]. If not treated, the abscess of the abdominal wall can eventually evolve into a cholecystocutaneous fistula (CCF). When the fistula drains at the skin, most of the tracts emerge into the right upper quadrant (48%) or the umbilicus (27%) [4]. However, some case reports locate it in more unusual locations such as the right hip and the gluteal region [5, 6]. When the fistula drains into an internal organ, duodenum, colon, stomach, it can manifest as a digestive fistula and biliary ileus [7–9]. There are also reports of fistulas draining at the uterus, kidney pelvis, portal vein, and ovarian cysts and with intrathoracic structures as pericardium or bronchial tree [10]. Gallstone ileus is a type of mechanical ileus involving impaction of one or more gallstones in the intestinal tract after they have migrated through a cholecystoenteric fistula. Nowadays, with the increasing reliability of diagnostic tools and treatments, the incidence of these complications is declining [11–18]. We report a case of a CCA in a 65-year-old woman who presented a painful mass in the right subcostal region. The diagnosis was made by computed tomography (CT). The treatment consisted of percutaneous drainage of the abdominal wall abscess followed by laparoscopic cholecystectomy in a two-stage protocol.

## 2. Case Presentation

**Figure 1:**
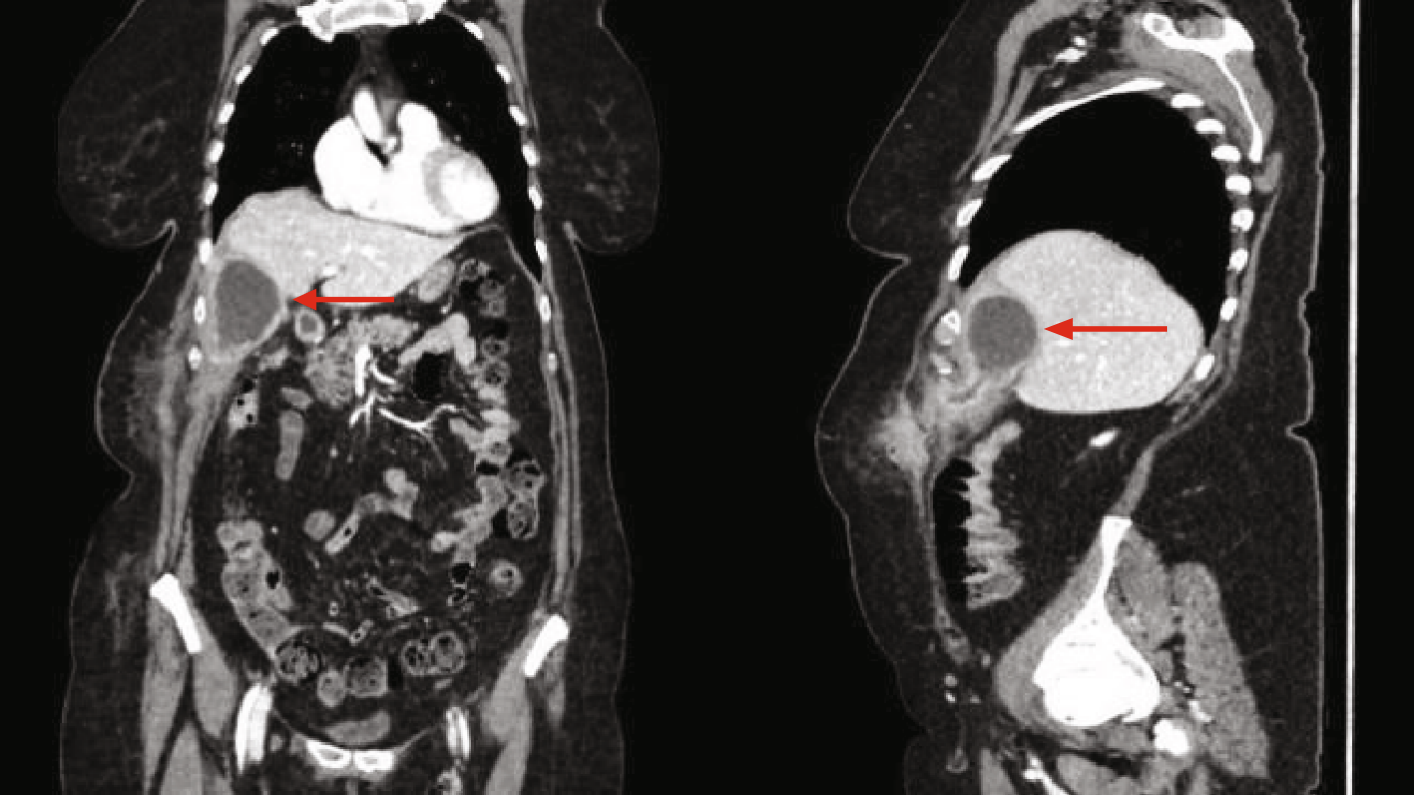
Abdominal computed tomography (CT) with the presence of a subparietal fluid collection of the right hypochondrium (40 × 45 × 65 mm).

A 65-year-old female, known only for obesity, was admitted to our department complaining of the right upper quadrant abdominal pain. The diagnosis of acute cholecystitis without complication was based on the clinical presentation, laboratory test, and ultrasound findings showing a lithiasic cholecystitis with impacted stones at the neck of the gallbladder. Due to diagnostic retard and in accordance with the patient, she was treated with oral antibiotics—ciprofloxacin and metronidazole—for 10 days and an elective laparoscopic cholecystectomy was programmed in a second time. Thereafter, she was readmitted due to the development of a mass in the right upper abdomen, increased in size with the time, with occasional pain but without fever. At the admission, the woman had normal vital signs and the clinical examination revealed a painful mass in right upper abdomen. Laboratory testing was notable for acute inflammation with elevated CRP (98 mg/l) (*N* < 5 mg/l) and hyperleukocytosis (15.4 G/l) (4.0 < *N* < 10.0 G/l) with normal liver function tests. An abdominal CT has demonstrated the presence of a subparietal collection of the right hypochondrium (40 × 45 × 65 mm) in communication with the gallbladder. That image showed off a perforated gallbladder with formation of an abscess and the presence of a second collection in the subcutaneous tissue connected by a fistula with the first abscess (Figure 1). The gallbladder was not distended, but it has slightly thickened walls.

**Figure 2:**
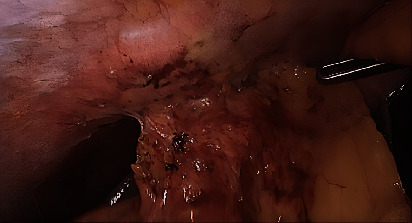
Intraoperative finding: chronic inflammation of the gallbladder to the abdominal wall.

**Figure 3:**
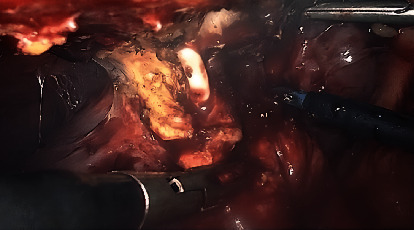
Percutaneous drainage catheter with fistula tract.

**Figure 4:**
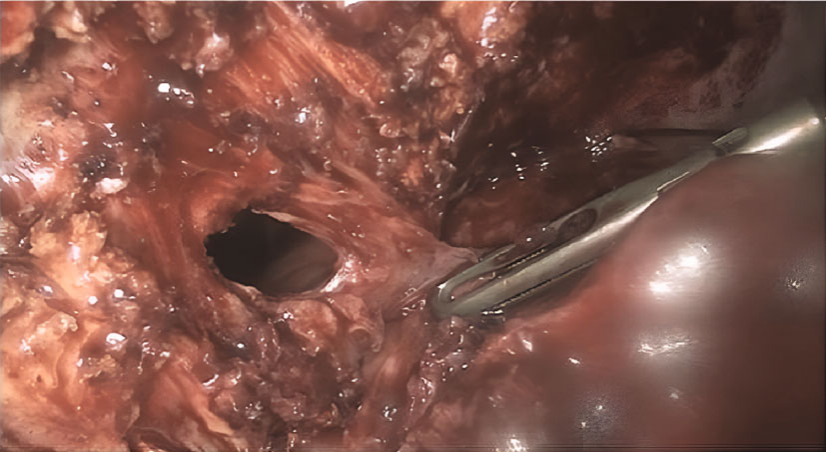
Abscess formation into the abdominal wall.

The liver was normal with a biliary and venous system permeable. The treatment consisted of 14 days antibiotic bitherapy—clindamycin and ceftriaxone with switch to ciprofloxacin and metronidazole—and the percutaneous drainage of the abscess. After percutaneous drainage, the liquid was analysed: cytology highlighted the presence of many gram-positive bacteria, the absence of bile pigments or malignant cells; microbiological culture was positive for Streptococcus anginosus and milleri, both sensible to penicillin. However, it was not possible to adapt the antibiotic due to a penicillin allergy of the patient. At 3 weeks of follow-up, the patient remains asymptomatic, with no residual abscess; the percutaneous drainage was still in place, with serous fluid leakage and an ultrasound confirmed the disappearance of the abscess. Laparoscopic cholecystectomy and the debridement of the abscess were performed 6 weeks later successfully. The intraoperative findings showed a chronic inflamed gallbladder, with full of adhesions between the gallbladder and the abdominal wall. After lysis of the adhesions, the percutaneous drainage catheter was found, showing the presence of a fistula tract draining the gallbladder into an abscess formation inside the abdominal wall and the diaphragm (Figures 2, 3, and 4). Anatomical pathology analysis found chronic inflammation and excluded malignancy. The postoperative follow-up was uneventful.

## 3. Discussion

This case demonstrates a very rare presentation of gallbladder perforation that created an abscess into the muscles of the abdominal wall. In the literature, most of the cases reported CCF [2], but there are not many cases presenting a CCA [11–18]. CCA is a result of neglected gallbladder disease [11]. CCA and CCF were historically a common outcome of gallbladder disease; now, they are rarely seen due to improvements in the identification and treatment of gallbladder pathology. There is no standard baseline management for this pathology, due to few numbers of cases and the differences in the quality of the patients' illness. However, this is a possible complication of gallbladder's pathology that all surgeons need to know. If the conditions of the patient are good, rather than perform immediate surgery that could lead to serious complications, we propose a two-stage approach. First is a percutaneous drainage of the abscess with placement of a catheter that will stay in place till the operation of cholecystectomy. The drain could be used for fistulography and cholangiography in case of suspicion of other fistula tracts. After the drainage, it will transform into an artificial CCF that now has to be managed as such. Secondly, after the active infection has subsided, the second intervention consists in a laparoscopic cholecystectomy with extraction of the catheter at the same time and debridement of the abscess' cavity. As 2020 WSES (World Society of Emergency Surgery) guidelines suggest for acute cholecystitis not operated in the first 10 days from the onset of symptoms [19], we recommend a delay of 6 weeks to perform the cholecystectomy after the drainage. In our opinion, this delay is reasonable. In addition, before performing the cholecystectomy, we advise confirming that the drainage of the abscess is complete, maybe by an image that may define anatomy before intervention, but should not delay surgery. The open cholecystectomy with excision of the fistulous tract is considered as a standard option for management [7]. In our opinion, the choice of open or laparoscopic cholecystectomy depends on the experience of the surgeon, on the absence of previous operations in the operative area, on the successful drainage of the abscess, and to a lesser extent on the comorbidities of the patient. The lack of adequate laparoscopic experience by the surgeon can lead to serious complications. The presence of comorbidities, like diabetes, malnutrition, old age, obesity, immunodeficiency, and steroid treatment can lead to failure of healing or to difficulty in performing surgery. For these reasons, a good analysis of a minimally invasive approach has to be done. When possible, as in this case report, the two-stage approach allows the surgeon to perform or at least attempt a minimally invasive approach. After drainage of the abscess, it is possible to perform cholecystectomy and the debridement of the abscess at the same time by laparoscopy and it is always possible to switch to an open procedure or to complete the management of the wall abscess externally. In case of failure of the conservative treatment, an adaptation of the antibiotic therapy has to be considered; a new drainage could be done, if needed. Even if with these changes the condition of the patient does not improve, an earlier cholecystectomy with drainage and debridement of the abscess has to be performed. The choice of open or laparoscopic cholecystectomy has to be decided based on the experience of the surgeon. However, in our opinion, laparoscopic management is the best to start with.

## 4. Conclusion

CCA is the result of neglected gallbladder disease. Nowadays, it is rarely seen due to improvements in the identification and treatment of gallbladder pathology. However, it is a possible complication of gallbladder's pathology that all surgeons have to know. Therefore, there is no standard baseline management for this pathology, due to the few numbers of cases and to the differences in the quality of the patients' illness. We propose a two-stage approach to manage this pathology. First is a percutaneous drainage of the abscess followed by the cholecystectomy, realised in a second time. The choice of open or laparoscopic cholecystectomy depends mostly on the experience of the surgeon. In our opinion, laparoscopic management is the best starting option, and just in case, the management of the wall abscess can be completed externally.

## Data Availability

Data are available on request.
